# Activity of Copper and Blast Furnace Slag and Its Influence on the Properties of Cement

**DOI:** 10.3390/ma19010038

**Published:** 2025-12-22

**Authors:** Stefania Grzeszczyk, Aneta Matuszek-Chmurowska, Alina Kaleta-Jurowska, Krystian Jurowski, Piotr Podkowa, Seweryn Stęplowski

**Affiliations:** 1 Faculty of Civil Engineering and Architecture, Opole University of Technology, Katowicka Street 48, 45-061 Opole, Poland; a.matuszek-chmurowska@po.edu.pl (A.M.-C.); a.kaleta-jurowska@po.edu.pl (A.K.-J.); k.jurowski@po.edu.pl (K.J.); 2 Odra Cement Plant, Budowlanych Street 9, 45-005 Opole, Poland; ppodkowa@odrasa.com.pl (P.P.); ssteplowski@odrasa.com.pl (S.S.)

**Keywords:** copper slag, blast-furnace slag, cement, pozzolanic activity, hydraulic activity, heat of hydration, strength

## Abstract

Reducing CO_2_ emissions from cement production is currently one of the major challenges faced by the cement industry. One approach to lowering these emissions is to reduce the clinker factor by incorporating alternative mineral additives into cement. Consequently, there is a growing interest in the use of copper slags (CSs) as supplementary cementitious materials. Therefore, this study investigates the properties of cements containing substantial amounts of copper slag (up to 60%) and, for comparison, the same proportions of granulated blast furnace slag. The inclusion of substantial amounts of CS results both from the lack of studies in this area and from the potential benefits associated with the utilization of larger quantities of copper slag. The chemical, phase, and particle size composition of CS and granulated blast furnace slag added to CEM I 42.5 cement from the Odra cement plant in amounts of 20%, 40%, and 60% by weight were compared. The pozzolanic activity index of the copper slag and the hydraulic activity index of the blast furnace slag were determined. The high pozzolanic activity of the CS was attributed to its high degree of vitrification (nearly 100%). In contrast, the lower hydraulic activity of the blast furnace slag was explained by its lower glass phase content (about 90% by mass). A gradual decrease in the total heat of hydration released within the first two days was observed with increasing slag content in the cement, slightly more pronounced for copper slags. However, at later stages (2–28 days), XRD analysis indicated higher hydration activity in cements containing copper slag, resulting from its strong pozzolanic reactivity. Cements with copper slag also showed slightly lower water demand compared to those with blast furnace slag. An increase in setting time was observed with higher slag content, more noticeable for blast furnace slag. The type and amount of slag in cement reduce both yield stress and plastic viscosity. Greater reductions were observed at higher slag content. Moreover, copper slag caused greater paste fluidity, attributed to the lower amount of fine particles fraction. The addition of slag decreased flexural and compressive strength in the early period (up to 7 days), this reduction being proportional to slag content. After 90 days, mortars containing 20% and 40% copper slag achieved strength values exceeding that of the reference mortar by 4%. In contrast, at a 60% CS content, a 5% decrease was observed, while for cement with 60% BFS the decrease was 11%. This indicates that a lower copper slag content in the cement (40%) is more favorable in terms of strength.

## 1. Introduction

The main factor driving the growing interest in copper slags is the significant increase in global copper production. According to the International Copper Study Group (ICSG), world copper production is expected to exceed 25 million tonnes by 2026, with demand continuing to rise. The rapid growth in copper production results in the generation of increasingly large amounts of copper slag waste. The global annual production of this type of slag is estimated to reach up to 60 million tonnes [1].

However, it must be noted that the use of copper slags may pose environmental and health risks due to their potential toxicity, primarily related to radioactivity and the presence of heavy metals. Therefore, they must be properly treated and processed to prevent any threat to the environment and human health. Considering the advantages of copper slags, their potential applications in the construction industry, particularly in cement manufacturing, are extensive.

Reducing CO_2_ emissions from cement production is currently one of the major challenges faced by the cement industry. The Emission Trading System (ETS) imposes a significant financial burden on the sector. According to the KOBiZE report [2] in January 2025 the price of CO_2_ emission allowances on the secondary market exceeded EUR 80 per tonne. Moreover, forecasts predict that CO_2_ emission prices may rise to as much as EUR 200 per tonne by 2030. This translates into a substantial increase in Portland cement production costs.

One of the ways to reduce these costs is to lower the clinker factor by using alternative mineral additives in cement (such as fly ash or granulated blast furnace slag) [3]. However, current climate policies assume a gradual phase-out of coal combustion in the energy sector, which will lead to reduced availability of these mineral additives. Consequently, there is growing interest in the use of copper slags for cement production [4,5,6].

Attempts to utilize copper slag as a cement additive were first undertaken in Poland in the 1970s. Research conducted at the AGH University of Science and Technology at that time confirmed the full suitability of copper slag as a cement component [7]. Between 1974 and 1975, several Polish cement plants, including Odra Cement Plant, experimented with producing cements containing copper slag—Portland cement with 15% slag, blast furnace cement with 30%, and other blast furnace cements with up to 45% copper slag [7].

In the current year, Odra Cement Plant has begun the production of CEM II/C-M (CS-LL) 32.5 R cement, containing 25% copper slag and 12% limestone.

Many authors associate the strength of cement-based materials with the pozzolanic properties of this slag. There are studies comparing the pozzolanic activity of copper slags with other mineral additives [8]. Numerous works focus on determining the optimal copper slag content in cement to achieve maximum material strength, which is usually around 30% [9,10,11].

There is also extensive research on the use of copper slag in concrete as both fine and coarse aggregate. Several studies have investigated the compressive and tensile strength of concrete specimens, which, according to the authors of [6,12,13,14], is comparable to or even higher than that of control mixes containing conventional sand and coarse aggregate. Aggregates derived from copper slags not only improve the strength of concrete but also reduce shrinkage, enhance resistance to sulfate attack [14], and increase the abrasion resistance of concrete [15].

Due to their chemical composition and high degree of vitrification, copper slags can serve as valuable pozzolanic additives to cement. As the energy sector moves away from coal combustion, copper slags could help balance the demand of the cement industry for mineral additives. Therefore, the cement industry is increasingly interested in the possibility of using this slag as a cement additive in larger quantities, particularly because it enables a reduction in CO_2_ emissions associated with Portland clinker production. Considering the above, studies were undertaken to evaluate the properties of cements containing substantial amounts of copper slag (up to 60%). Until now, research has focused on cements with slag contents of up to 40%. The present investigation was conducted in cooperation with Cementownia Odra, a producer of blast furnace slag cement (CEM III) containing approximately 60% granulated blast furnace slag. This fact also inspired a comparative study of the properties of slag cements containing equal amounts (up to 60% by mass) of blast furnace slag and copper slag.

Stored copper slags may pose an environmental threat due to the presence of heavy metals, which can leach into water and soil [16,17,18]. For this reason, numerous studies and industrial efforts are aimed at recovering heavy metals such as iron, copper, cobalt, and nickel from copper slags [19,20,21,22]. Most research focuses on copper recovery [23]; nevertheless, significant quantities of copper slag remain stockpiled without processing for heavy metal extraction.

Using copper slags as a cement additive significantly reduces the leachability of heavy metals from cement-based materials (primarily concrete) containing such slags. It has been shown that heavy metal leachability decreases with an increase in pH [24]. Moreover, in the high-pH environment of cement paste, heavy metals transform into forms of reduced solubility and may be incorporated into hydration products. Considering that cement typically constitutes 10–20% by mass of ordinary concrete, the concentration of heavy metals in this material will be proportionally lower.

While prior research mainly evaluated copper slag at replacement ratios ≤ 40%, this study extends the replacement to 60% and provides a direct comparison against BFS-based commercial compositions. The high replacement of clinker is relevant from an ecological point of view, as it significantly reduces carbon dioxide emissions per ton of cement. The use of copper slag in cement production is also economically justified, because it is a waste material that accumulates in landfills in considerable quantities. The present study aims to compare the properties of copper slag and blast furnace slag used in cement production at Odra Cement Plant (Opole, Poland), as well as to investigate the characteristics of slag cement containing up to 60% by mass of granulated blast furnace slag and copper slag.

## 2. Characteristics of the Tested Slags

### 2.1. Particle Size Distribution

**Figure 1:**
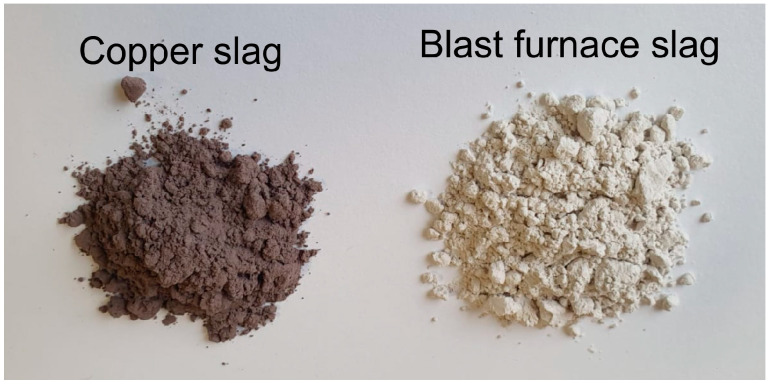
Sample of copper slag and blast furnace slag used in investigation.

**Figure 2:**
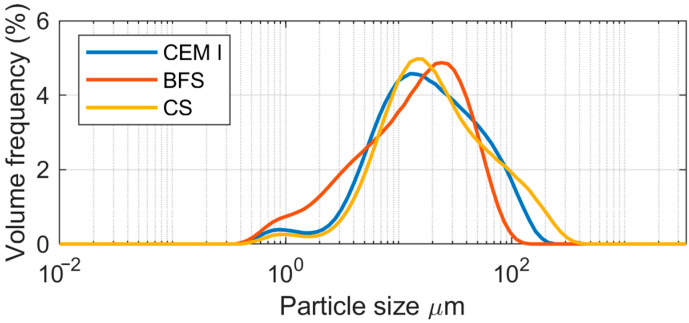
Differential particle size distribution curve for CEM I, BFS, and CS [25].

For the study, copper slag was ground in a laboratory ball mill to achieve a specific surface area comparable to that of granulated blast furnace slag (BFS), which had been ground in an industrial roller-bowl mill at the Odra Cement Plant. The materials are shown in Figure 1. The results of the particle size distribution analysis for the cement used in the study (CEM I 42.5 R from Odra Cement Plant, designated as CEM I), granulated blast furnace slag (BFS), and copper slag (CS) are presented in Figure 2 and Table 1.

The analysis of the results indicates that the granulated blast furnace slag is slightly more finely ground than the copper slag. BFS is characterized by a slightly higher specific surface area compared to CS and contains the largest proportion of fine particles among the materials tested (Table 1). In this slag, 10% of the particles have a diameter below 2.6 µm, and 50% have a diameter smaller than 15.9 µm.

The results of the specific density measurements show that the copper slag has a density of 3.09 g/cm^3^, which is slightly higher than that of the blast furnace slag (2.99 g/cm^3^). The specific density of copper slags can, however, be significantly higher—up to 3.9 g/cm^3^ [6]. The authors of [25] relate the density of copper slag to its iron content, which may range from 30 to 60 wt.% [6,26].

### 2.2. Chemical and Phase Composition

The results of the chemical composition analysis of CEM I, BFS, and CS are presented in Table 2. A comparison of the chemical compositions shows that copper slag contains significantly less CaO than granulated blast furnace slag (24.1 wt.% vs. 43.8 wt.%) and a much higher amount of Fe_2_O_3_ (17.4 wt.% compared to only 0.5 wt.% in BFS).

Based on their chemical composition, copper slags are classified as acidic slags and are generally considered non-hydraulically active. In contrast, granulated blast furnace slags belong to the group of slightly alkaline slags. When they contain a high proportion of the glassy phase (above 80%), they exhibit good hydraulic activity [27,28,29].

**Figure 3:**
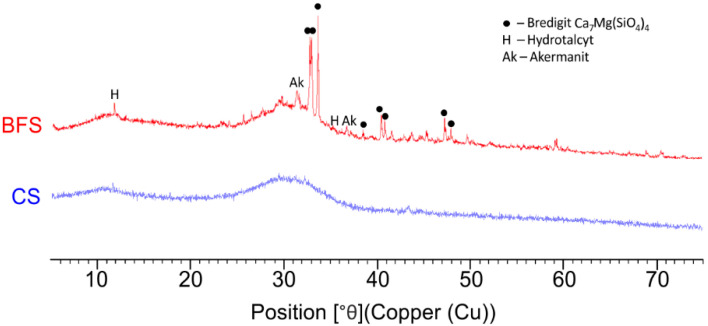
X-ray diffraction patterns of copper slag and granulated blast furnace slag [30].

The XRD of copper slag (Figure 3) shows no reflections from crystalline phases, while the characteristic increase in the background within the 2θ angle range from 26° to 38° indicates a high degree of glass transition in the copper slag. This is confirmed by the determined glassy phase content, which is approximately 100% by mass. In turn, the diffraction pattern of granulated blast furnace slag shows quite intense diffraction lines belonging to the crystalline phases present in the slag: akermanite, hydrotalcite, and bredigitite. This indicates a lower degree of glass transition in the slag. The determined glassy phase content in granulated blast furnace slag is approximately 90% by mass and is lower than the glassy phase content in copper slag.

### 2.3. Hydraulic and Pozzolanic Activity of the Slags

The activity of slag depends on numerous factors, primarily its chemical and mineral composition, the amount of glassy phase, and the degree of fineness [31,32,33]. The relationship between the chemical and physical characteristics of BFS and its hydraulic activity has been widely discussed [34,35]. Despite these discussions, several empirical equations (moduli) are used to relate hydraulic activity to chemical composition, particularly the ratio of alkaline to acidic oxides.

In Poland, Japan, and Germany, the most commonly used parameter is the activity coefficient defined as Z = (CaO + MgO + Al_2_O_3_)/SiO_2_. For Polish slags, Z typically ranges from 1.35 to 1.55 [33]. The Z-value for the BFS used in this study is 1.45.

Because of the complex influence of the chemical and phase composition of BFS on its hydraulic activity, this property is also determined by directly measuring the effect of ground BFS on the compressive strength of mortars containing a mixture of Portland cement and BFS [33,36]. The hydraulic activity index value determined in this way is presented in Table 3.

The results show that the hydraulic activity index of BFS after 28 days is 88.2%, meeting the standard requirements. Comprehensive studies on the influence of the glassy phase content in BFS on the properties of slag cements were carried out by the authors of [37]. They demonstrated that the degree of vitrification has the greatest effect on hydraulic activity: when the glassy phase content approaches 100%, the hydraulic activity index reaches 108.4% after 28 days, while for a glassy phase content of about 90%, the value drops to 88.8%, corresponding to approximately 10 MPa lower compressive strength of the mortar after 28 and 90 days. The BFS tested in this study contains about 90% glassy phase and shows a hydraulic activity index of 88.2% after 28 days, consistent with the results reported in [37]. Increasing the glassy phase content in BFS would therefore enhance its hydraulic activity.

Due to their chemical composition and high degree of vitrification, copper slags can serve as pozzolanic additives in cement production. Although they are generally regarded as non-hydraulic materials [38]. Distinguishing between pozzolanic and hydraulic components can be challenging. Wesche [39] proposed that this distinction can be based on the CaO/SiO_2_ ratio: when the ratio is below 0.5, the material exhibits pozzolanic activity, while higher values indicate hydraulic activity, increasing with the CaO/SiO_2_ ratio.

The amount of reactive silica in the copper slag is 33.5 wt.%, which meets the requirements of EN 197-1 [40], specifying a minimum of 25 wt.% for pozzolanic materials. The pozzolanic activity index of copper slag, determined in accordance with [41] was measured directly by evaluating the compressive strength of mortars containing mixtures of Portland cement and copper slag.

The results show that the pozzolanic activity index of copper slag reaches 99% after 28 days and 110.5% after 90 days, significantly exceeding the standard requirements [41]. These values confirm the high pozzolanic reactivity of copper slag. For comparison, the pozzolanic activity indices of siliceous fly ashes used in Polish cement production typically range from 80 to 90% after 28 days and 90 to 100% after 90 days [42].

## 3. Research Methods

The particle size distribution was determined using a Malvern Mastersizer 3000 laser particle size analyzer (Malvern Panalytical Ltd., Malvern, UK). Isopropanol was used as the dispersing medium for cement, while distilled water was used for slags. The volume particle size distribution was presented as both a differential and a cumulative curve.

X-ray diffraction (XRD) analyses were carried out using a modern PANalytical X’Pert Pro diffractometer PW3040/60 (PANalytical, Almelo, The Netherlands). The analyses were performed over a wide 2θ range (5–65°) to obtain an appropriate number of diffraction peaks for each phase, with a constant long counting time (300 s) and a precise step size (0.02° 2θ).

The hydraulic activity index of the granulated blast furnace slag (BFS) was determined in accordance with EN 15167-1:2007 [43]. The activity index was calculated as the percentage ratio of the compressive strength of a binder composed of 50% ground BFS and 50% cement to the compressive strength of the reference cement (CEM I 42.5R).

The pozzolanic activity index of the copper slag (CS) was determined in accordance with EN 450-1:2012 [41]. The activity index was calculated as the percentage ratio of the compressive strength of a binder composed of 25% copper slag and 75% cement to that of the reference cement (CEM I 42.5R).

The rheological properties of the cement pastes were tested using a RHL HAAKE MARS III rotational rheometer with coaxial cylinders (Thermo Fisher Scientific, Waltham, MA, USA). The same sample preparation procedure and identical measurement conditions were applied to all tested pastes. During the measurements, the shear rate was uniformly increased from 0 to 150 s^−1^ over 180 s, and then decreased from 150 s^−1^ to 0 over the next 180 s. The first measurement was taken 10 min after mixing cement with water, and subsequent measurements were repeated after 30 min and 60 min. All tests were carried out at a temperature of 20 ± 0.1 °C with a water-to-cement ratio (*w*/*c*) of 0.4.

The standard consistency, water demand, and initial and final setting times of the binders were determined according to EN 196-3 + A1:2011 [44]. These tests were performed after determining water demand, maintaining standard consistency throughout the procedure.

The heat of hydration of the binders was measured using a SETARAM C-80 isothermal microcalorimeter (SETARAM, Caluire-et-Cuire, France). The same procedure for preparing the analytical samples and identical test conditions were applied for all cement pastes: temperature 21 ± 1 °C and *w*/*c* = 0.4. The results were presented as plots of the rate and total heat evolution over time.

The compressive and flexural strengths of mortars were determined in accordance with PN-EN 196-1:2016-07 [45], using specimens of dimensions 40 mm × 40 mm × 160 mm. The specimens were demolded after 24 h and cured in water at 20 ± 2 °C until testing.

**Figure 4:**
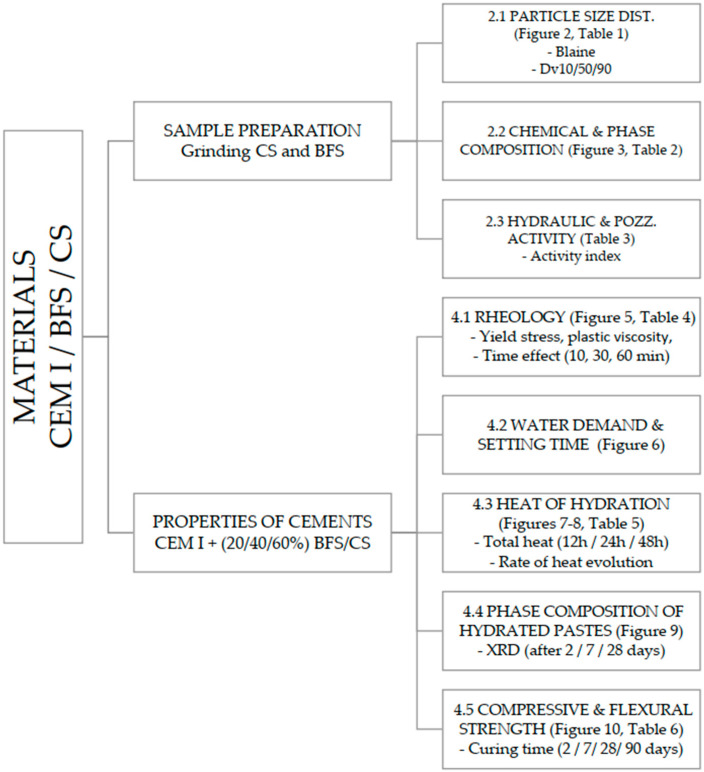
A methodological scheme of investigation.

A methodological scheme of investigation is presented in Figure 4.

## 4. Properties of Slag Cements

### 4.1. Rheological Properties

**Figure 5:**
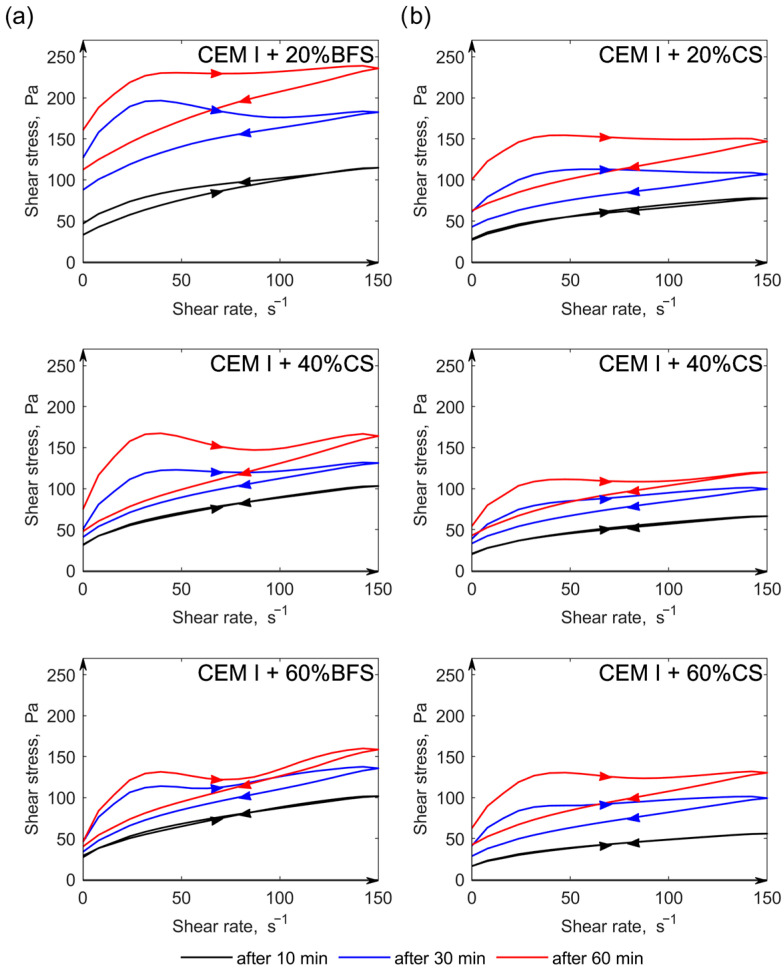
Flow curves of cement pastes: CEM I with 20%, 40%, and 60% by mass of BFS (**a**) and CS (**b**), after 10, 30, and 60 min.

Slag cements were obtained by homogenizing CEM I 42.5 R Portland cement from Odra Cement Plant with granulated blast furnace slag (BFS) and copper slag (CS) in the amounts of 20%, 40%, and 60% by mass of each component in the cement. The yield stress and plastic viscosity values are presented in Table 4, while the flow curves are shown in Figure 5.

The rheological tests clearly indicate that the incorporation of slags into cement results in a reduction in yield stress, which becomes more pronounced with increasing slag content. Moreover, pastes containing copper slag exhibit lower yield stress compared to those containing the same amount of blast furnace slag. Similarly, pastes with copper slag are characterized by lower plastic viscosity than those with BFS. However, as the copper slag content increases from 20% to 60% by mass, the plastic viscosity decreases at early hydration stages (10 min) and increases at later times (30 and 60 min). In contrast, increasing the amount of BFS in cement leads to higher viscosity after 10 and 30 min, followed by a decrease after 60 min. The obtained rheological test results for pastes containing copper slag are consistent with the findings of the authors of study [10] who also reported a decrease in rheological parameters (yield stress and plastic viscosity) with increasing slag content in the cement.

Over time (from 10 to 60 min), an increase in rheological parameters (yield stress and plastic viscosity) was observed in all pastes containing both types of slag. The effect of time on these parameters was significantly greater than that of slag content in cement in the range of 20–60% by mass. Furthermore, analysis of the hysteresis loop area of the flow curves revealed that, with time, all slag-containing pastes exhibited an increasing degree of thixotropy, regardless of the slag type.

The higher degree of fluidity observed in pastes with copper slag, compared to those with BFS, can be attributed to the lower fineness of the copper slag (smaller specific surface area and fewer fine particles). Similar effects of fine particle content on the rheological properties of slag have been reported by other authors [10,46,47].

As reported by [48], the yield stress and plastic viscosity of pastes containing BFS may vary (increase or decrease) depending on the ratio of specific surface areas of the cement and slag. However, many factors influence the rheological properties of slag-containing pastes — including the percentage of addition, particle size and shape, and particularly the cement composition, especially C_3_A content [49,50].

According to the study [10] the shape of copper slag particles has a particularly strong influence on the yield stress, increasing the cohesive forces within the cement paste. However, accounting for the effect of copper slag particle shape on rheological properties requires further investigation.

### 4.2. Water Demand and Setting Time

**Figure 6:**
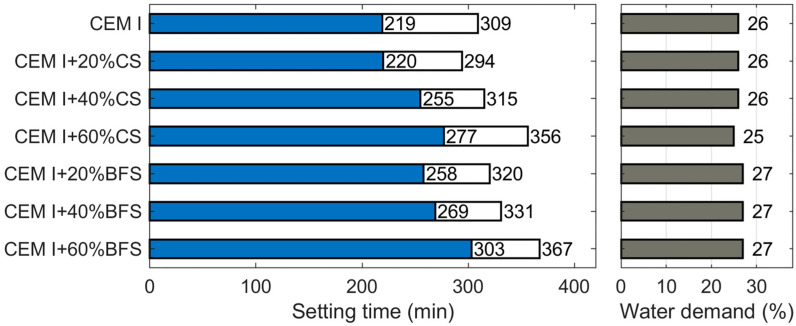
Water demand and setting times of CEM I containing 20%, 40%, and 60% by mass of CS and BFS.

The results of standard consistency tests for slag cements showed that cements containing CS have a slightly lower water demand (25–26%) than those containing BFS (27%), and this value remains almost unchanged with increasing slag content (20–60% by mass). Increasing the CS content to 60% by mass reduced the water demand from 26% to 25% (Figure 6). The lower water demand of CS-blended cement is associated with the smaller specific surface area and lower proportion of fine particles in copper slag (Table 1). According to [51] the reduced water demand of CS-blended cements results from the higher density and lower open porosity of copper slag compared to BFS.

The results of setting time tests (Figure 6) clearly show that both BFS and CS extend the setting time (initial and final) compared to the reference cement. The prolongation is more pronounced with increasing slag content and is slightly greater for BFS. The delayed setting of BFS-blended cement is a well-known phenomenon [33]. and similar effects have also been observed for CS [6,52].

### 4.3. Heat of Hydration

The effect of BFS and CS on the hydration kinetics of cement at early stages was determined using isothermal microcalorimeter.

**Figure 7:**
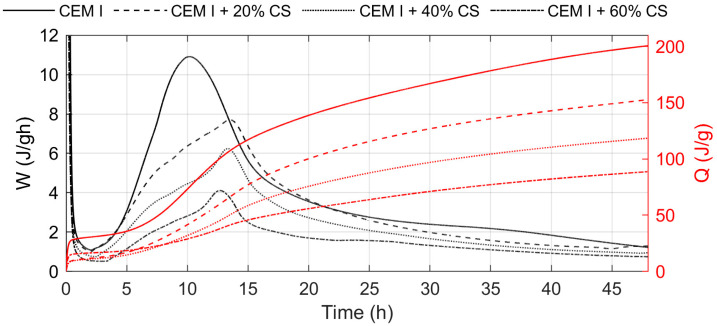
Rate and total heat evolution during hydration of cement containing 20%, 40%, and 60% by mass of CS.

**Figure 8:**
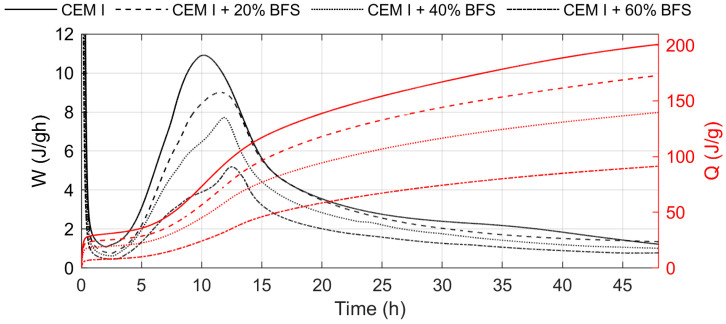
Rate and total heat evolution during hydration of cement containing 20%, 40%, and 60% by mass of BFS.

The results of the heat evolution rate tests, for cement pastes containing 20%, 40%, and 60% CS are shown in Figure 7, while the corresponding results for BFS-blended cements (at the same replacement levels) are presented in Figure 8. The total heat release during hydration for all cements is summarized in Table 5.

As shown in Figure 7 and Figure 8, the addition of slags (both copper and blast furnace) to cement causes a delay in the occurrence of the peak corresponding to the hydration of tricalcium silicate (C3S) compared to the reference cement. This delay is more pronounced in the presence of copper slag. Increasing the slag content from 20% to 60% by mass only slightly affects the position of this peak. The reduced intensity of the silicate peak with increasing slag content results from the lower amount of C3S phase in the blended cement.

The total heat released during hydration after 12, 24, and 48 h was lower for cements containing either copper or blast furnace slag compared to plain cement (Table 5). Furthermore, a higher slag content caused a significant decrease in the total released heat. A similar reduction in heat evolution for cements with copper slag has also been reported by other authors [1,10]. The lower heat evolution observed for cements containing CS compared to those with BFS indicates differences in hydration activity of cement in the presence of different kind of slag.

The relatively low heat of hydration of cement containing copper slag during the early stage reflects the pozzolanic nature of the reaction, which becomes more pronounced at later ages. Moreover, the lower fineness of the CS may also contribute to the reduced amount of heat released during the initial hydration period. The results of the heat of hydration tests are consistent with the findings reported by other authors [10,53].

### 4.4. Phase Composition of Hydrated Pastes

**Figure 9:**
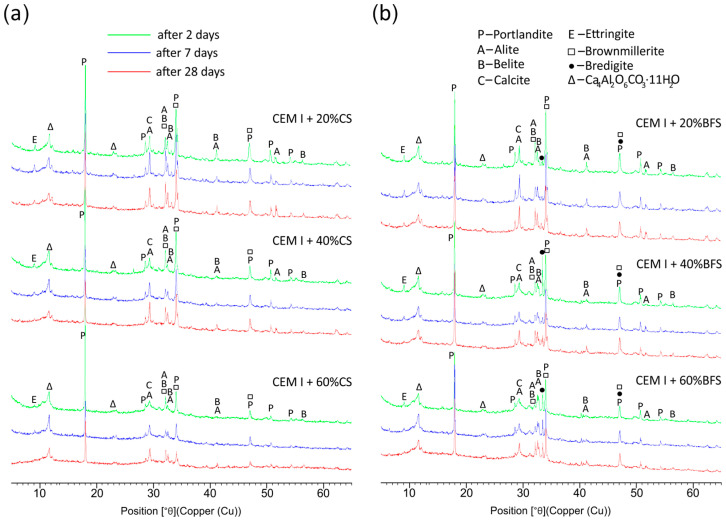
XRD patterns of pastes made with CEM I containing 20%, 40%, and 60% by mass of CS (**a**) and BFS (**b**) after 2, 7, and 28 days of hydration.

The XRD patterns of pastes containing 20%, 40%, and 60% by mass of copper slag (CS) and blast furnace slag (BFS) after 2, 7, and 28 days of hydration are shown in Figure 9. All diffractograms display diffraction lines corresponding to clinker phases—alite, belite, and brownmillerite—as well as hydration products, such as portlandite and ettringite. Additionally, the patterns of pastes containing BFS show diffraction lines of bredigite, a crystalline phase present in the blast furnace slag.

Over time (after 7 and 28 days), the intensities of the diffraction peaks corresponding to alite and belite decrease, as does the intensity of the portlandite peak—the latter diminishing more strongly with increasing slag content (both CS and BFS). Analysis of the main diffraction line of portlandite (d = 2.628 Å) shows that this decrease is more pronounced in pastes containing copper slag. This indicates a greater consumption of Ca(OH)_2_ in this environment (Figure 9). The mechanism of the pozzolanic reaction in the highly alkaline environment of a cement paste is well understood. The glassy phase present in pozzolans is considered a reactive component in the cement paste. Pozzolanic constituents undergo hydrolysis, during which silicon and aluminum ions are released into the liquid phase of the paste, where they react with calcium ions to form hydrated calcium silicate phases and hydrated calcium aluminate phases. The resulting C–S–H phase exhibits a lower C/S ratio than that formed in Portland-cement paste [33]. The presence in concrete of a C–S–H phase with a lower C/S ratio enhances the durability of the material [54]. For these reasons, among others, the use of reactive copper slags as a mineral additive to cement is recommended. The higher consumption of Ca(OH)_2_ is also confirmed by the strength test results (Table 6), which indicate that the strength gains are higher for cements containing CS.

The gradual decrease in the main portlandite diffraction peak with increasing slag content (from 20% to 60% by mass) can also result from the reduced cement content in these blended samples.

### 4.5. Compressive and Flexural Strength

**Figure 10:**
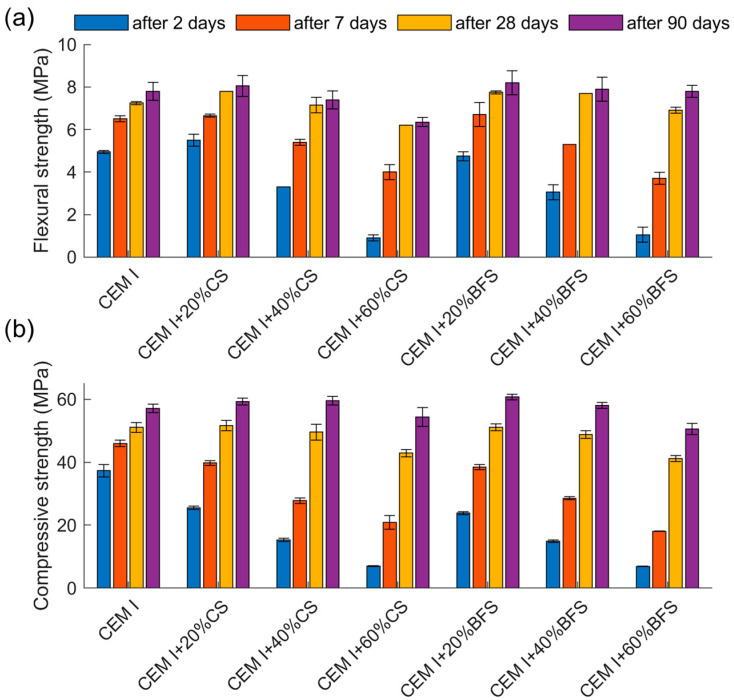
Compressive and flexural strength of mortars containing 20%, 40%, and 60% by mass of CS and BFS after 2, 7, 28, and 90 days of curing: (**a**) flexural strength; (**b**) compressive strength.

The results of compressive and flexural strength tests of mortars made of cement without mineral additives and of cement containing 20%, 40% and 60% by mass of CS and BFS after 2, 7, 28 and 90 days of maturation are presented in Table 6 and Figure 10.

As expected, cements containing slags exhibit a slower strength development rate during the early stages of hardening, particularly within the first 7 days. However, after 7 days, due to the high pozzolanic activity in the case of CS and hydraulic activity in the case of BFS—the strength gain becomes significantly greater. After 28 days of curing, cement containing 20% and 40% slag (CS and BFS) reach compressive strengths comparable to that of the reference mortar.

After 90 days, mortars containing 20% and 40% of CS or BFS achieve higher compressive strengths than the reference sample (57.1 MPa), reaching values between 58.1 and 60.7 MPa (102–104% of the reference value). For mortars with 60% slag content, a reduction in compressive strength is observed compared to the reference—by about 5% for CS and 11% for BFS. The development of flexural strength over time follows a similar trend to that of compressive strength. Influence of the pozzolanic properties of copper slags on the strength and characteristics of binding materials has also been observed by other authors [8,55,56,57]. The authors of the studies [8,58,59] indicate that both mechanical activation (increasing the degree of fineness) and alkaline activation are effective methods for enhancing the reactivity of copper slags, which results in improved material strength. Nevertheless, these studies reported a lower increase in strength than that observed by the authors of the present work, indicating that the copper slag used in the present investigations exhibited higher reactivity.

Since a decrease in compressive strength was noted at cement containing 60% in comparison to 40% slag, it is advisable to conduct studies aimed at determining the optimal copper slag content in cement within this range.

Analysis of flexural strength results also indicates that a small addition of slag (20%) slightly increases the flexural strength compared to mortars with higher slag contents (40% and 60%). The significant strength gains observed at later ages (7–90 days) are consistent with XRD results, confirming the high pozzolanic activity of copper slag and hydraulic activity of blast furnace slag.

## 5. Conclusions

The pozzolanic activity index of the investigated copper slag, determined based on direct measurements of its effect on mortar strength, is high, reaching 99% after 28 days and 110% after 90 days. These values are even slightly higher than those typically obtained for siliceous fly ashes used in Poland. The high pozzolanic activity of copper slag is primarily attributed to its very high glass content, approximately 100% by mass.

The obtained values of the hydraulic activity index for blast furnace slag—55.0% after 7 days and 88.2% after 28 days—result from its lower glassy phase content (about 90%).

The heat of hydration tests of slag cements revealed a delay in the main heat release peak associated with the hydration of tricalcium silicate and a reduction in the total heat evolved during the first two days. Increasing the slag content caused a gradual decrease in the intensity of the C_3_S hydration peak and a reduction in total heat evolution, which was more pronounced for cements containing copper slag.

X-ray diffraction analysis of the hydration process (from 2 to 28 days) showed slightly higher hydration activity for cement containing copper slag. This was evidenced by a more significant decrease in the diffraction intensities of the clinker phases (alite, belite, and brownmillerite) and, particularly, by the reduction in the main portlandite peak (d = 2.628 Å).

Cements containing the same proportions of copper slag (CS) and blast furnace slag (BFS) exhibited similar properties, including comparable density (3.09 and 2.99 g/cm^3^, respectively) and water demand (25–26% for CS and 27% for BFS) for slag contents between 20% and 60%. The setting time increased with higher slag content in both cases, with a slightly greater delay observed for BFS.

The rheological tests demonstrated that the addition of slags (CS and BFS) to cement led to a decrease in yield stress and plastic viscosity, which became more pronounced with increasing slag content. The lower rheological parameters (greater fluidity) observed for cement pastes containing copper slag were attributed to the lower content of fine particles in this material.

The comparison of compressive and flexural strengths of mortars containing copper and blast furnace slag showed that, in the early curing period, mortars with slag exhibited lower strength than the reference mortar. The reduction was greater at higher slag content. However, after 90 days, mortars containing 20% and 40% of slag achieved higher compressive strengths than the reference sample. This behavior reflects the high pozzolanic activity of copper slag and the hydraulic activity of blast furnace slag.

In the case of cements containing 60% slag, a decrease in compressive strength after 90 days of curing is observed compared to the reference cement. This indicates that a lower slag content (20–40%) is more favorable in terms of strength.

In summary, copper slag can be considered a valuable supplementary cementitious material and a promising alternative to granulated blast furnace slag. For strength-related reasons, the recommended proportion of CS in cement is 20–40%. Since a decrease in compressive strength was observed in cement containing 60% CS compared to 40% CS (approximately 9%), higher cement replacement levels may still be considered from an economic and ecological perspective, provided that the copper slag meets the requirements regarding radioactivity and heavy metal content. This will ensure the protection of the natural environment, and, in particular, human health.

## Figures and Tables

**Table 1:** Blaine specific surface area and Dv(10), Dv(50), Dv(90) values for CEM I, BFS, and CS [25].

Material	Blaine Surface Area (m^2^/kg)	Dv(10) (µm)	Dv(50) (µm)	Dv(90) (µm)
CEM I	448.0	5.1	18.4	74.4
BFS	410.0	2.6	15.9	47.9
CS	400.0	6.2	20.4	103.0

**Table 2:** Chemical composition of CEM I, BFS, CS (wt.%) [25].

Material	SiO_2_	Al_2_O_3_	Fe_2_O_3_	CaO	MgO	K_2_O	Na_2_O	SO_3_
CEM I	19.6	6.4	2.7	63.5	4.1	0.8	0.2	2.4
BFS	39.3	7.9	0.5	43.8	5.1	0.4	0.5	0.2
CS	33.5	12.2	17.4	24.1	5.5	3.0	1.5	0.1

**Table 3:** Hydraulic activity index of BFS and pozzolanic activity index of CS [30].

**Slag**	**Hydraulic Activity Index, Over Time** **(%)**			
	7 Days	Requirements	28 Days	Requirements
BFS	55.0	≥45.0	88.2	≥70.0
	**Pozzolanic activity index, over time** **(%)**			
	28 days	Requirements	90 days	Requirements
CS	99.0	≥75.0	110.5	≥85.0

**Table 4:** Yield stress and plastic viscosity of pastes made with CEM I and CEM I containing 20%, 40%, and 60% by mass of CS and BFS.

Cement Type	Yield Stress (Pa)			Plastic Viscosity (Pa·s)		
	After Time (min)					
	10	30	60	10	30	60
CEM I	73	104	126	0.28	0.48	0.65
CEM I + 20%BFS	70	113	134	0.32	0.50	0.73
CEM I + 40%BFS	51	63	65	0.38	0.49	0.67
CEM I + 60%BFS	47	56	61	0.40	0.54	0.66
CEM I + 20%CS	42	58	76	0.25	0.34	0.47
CEM I + 40%CS	33	48	61	0.24	0.36	0.43
CEM I + 60%CS	28	42	58	0.21	0.40	0.49

**Table 5:** Total heat evolution during hydration of cement: CEM I and CEM I + 20%, 40%, and 60% CS and BFS.

Cement Type	Total Heat of Cement Hydration After Time (J/g)		
	12 h	24 h	48 h
CEM I	94.2	151.5	200.7
CEM I + 20%BFS	74.3	130.2	172.9
CEM I + 40%BFS	59.4	104.5	139.7
CEM I + 60%BFS	32.8	65.6	91.4
CEM I + 20%CS	55.0	112.9	152.8
CEM I + 40%CS	41.5	85.5	118.5
CEM I + 60%CS	35.3	62.2	88.7

**Table 6:** Compressive and flexural strength of mortars containing granulated blast furnace slag and copper slag.

Cement Type	Flexural Strength (MPa)				Compressive Strength (MPa)			
	After Time (Days)				After Time (Days)			
	2	7	28	90	2	7	28	90
CEM I	5.0	6.5	7.3	7.8	37.3	46.0	51.1	57.1
CEM I + 20%CS	5.5	6.7	7.8	8.1	25.5	39.8	51.7	59.3
CEM I + 40%CS	3.3	5.4	7.2	7.4	15.3	27.8	49.6	59.6
CEM I + 60%CS	0.9	9.6	6.2	6.4	7.0	20.9	42.9	54.3
CEM I + 20%BFS	4.8	6.7	7.8	8.2	23.8	38.5	51.2	60.7
CEM I + 40%BFS	3.1	5.3	7.7	7.9	14.9	28.6	48.8	58.1
CEM I + 60%BFS	1.1	3.7	6.9	7.8	6.9	18.1	41.2	50.6

## Data Availability

The original contributions presented in this study are included in the article. Further inquiries can be directed to the corresponding author.
